# Amelogenesis imperfecta caused by N-terminal enamelin point mutations in mice and men is driven by endoplasmic reticulum stress

**DOI:** 10.1093/hmg/ddx090

**Published:** 2017-03-11

**Authors:** Steven J. Brookes, Martin J. Barron, Claire E.L. Smith, James A. Poulter, Alan J. Mighell, Chris F. Inglehearn, Catriona J. Brown, Helen Rodd, Jennifer Kirkham, Michael J. Dixon

**Affiliations:** 1 Department of Oral Biology, School of Dentistry, Wellcome Trust Brenner Building University Of Leeds, St James's University Hospital, Leeds LS9 7TF, UK; 2 Faculty of Biology, Medicine & Health, Manchester Academic Health Sciences Centre, University of Manchester, Michael Smith Building, Manchester M13 9PT, UK; 3 Department of Oral Medicine, School of Dentistry, University of Leeds, Leeds, UK; 4 Leeds Institute of Biomedical and Clinical Sciences, St James’s University Hospital, University of Leeds, Leeds LS9 7TF, UK; 5 Birmingham Dental Hospital and School of Dentistry, Birmingham B5 7EG, UK; 6 Unit of Oral Health and Development, School of Clinical Dentistry, University of Sheffield, Sheffield, UK

## Abstract

‘*Amelogenesis imperfecta’* (AI) describes a group of inherited diseases of dental enamel that have major clinical impact. Here, we identify the aetiology driving AI in mice carrying a p.S55I mutation in enamelin; one of the most commonly mutated proteins underlying AI in humans. Our data indicate that the mutation inhibits the ameloblast secretory pathway leading to ER stress and an activated unfolded protein response (UPR). Initially, with the support of the UPR acting in pro-survival mode, *Enam*^p.S55I^ heterozygous mice secreted structurally normal enamel. However, enamel secreted thereafter was structurally abnormal; presumably due to the UPR modulating ameloblast behaviour and function in an attempt to relieve ER stress. Homozygous mutant mice failed to produce enamel. We also identified a novel heterozygous *ENAM*^p.L31R^ mutation causing AI in humans. We hypothesize that ER stress is the aetiological factor in this case of human AI as it shared the characteristic phenotype described above for the *Enam*^p.S55I^ mouse. We previously demonstrated that AI in mice carrying the *Amelx*^p.Y64H^ mutation is a proteinopathy. The current data indicate that AI in *Enam*^p.S55I^ mice is also a proteinopathy, and based on comparative phenotypic analysis, we suggest that human AI resulting from the *ENAM^p.L31R^* mutation is another proteinopathic disease. Identifying a common aetiology for AI resulting from mutations in two different genes opens the way for developing pharmaceutical interventions designed to relieve ER stress or modulate the UPR during enamel development to ameliorate the clinical phenotype.

## Introduction

Mature dental enamel, which is composed of approximately 96% mineral and 4% organic material and water compared to approximately 70% mineral and 30% organic material and water found in dentine and bone, represents the most extreme form of mammalian biomineralization. Enamel mineral is a substituted hydroxyapatite (HA) and is the hardest and most resilient of the skeletal tissues (1). The wear resistance of dental enamel is a consequence of its highly ordered structure; the mineral crystallites are organized into interlocking prismatic, or rod, structures interspersed with interprismatic crystallites (1,2). Amelogenesis, the formation of dental enamel, is presumed to be mediated by the interactions of enamel extracellular matrix proteins coupled with the regulated deposition of mineral (3). Amelogenesis occurs in two broad stages and is mediated by a specialized epithelium comprised of ameloblast cells (3). In the early, secretory stage of amelogenesis, the ameloblasts elaborate the enamel extracellular matrix proteins composed principally of amelogenin (>90%), ameloblastin and enamelin (3). Post-secretory enzymatic processing of the matrix proteins leads to the self-directed organization of the enamel extracellular matrix and delineates the three-dimensional structure of the nascent enamel (3). Once the full thickness of the enamel has been deposited, the ameloblasts begin to degrade the enamel extracellular organic matrix and subsequently increase transport of mineral ions into the forming enamel (3). Removal of the proteinaceous matrix allows the initial HA crystallites to grow laterally until the full mineral content of the tissue is achieved (3).

Inherited defects of dental enamel biomineralization, *amelogenesis imperfecta* (AI; MIM PS104500), constitute a common group of genetic diseases that occur with an incidence as high as 1:700 live births (4). Affected individuals frequently experience severe problems with self-esteem due to the appearance of their teeth as well as pain due to sensitivity and require substantial and often complex dental care to manage the condition (5). The critical role played by the proteins of the extracellular matrix in enamel biomineralization is highlighted by the discovery that many of the genes mutated in AI encode matrix proteins or the enzymes that modify them (6,7). For example, mutations in the amelogenin (*AMELX*) gene underlie X-linked forms of AI (MIM #301200), while mutations in the gene encoding enamelin (*ENAM*) are associated with both autosomal dominant and autosomal recessive AI phenotypes (MIM #104500 and #204650) (6,7). The importance of enamelin for normal amelogenesis is clear because *ENAM* mutations are relatively common in AI probands causing predominantly autosomal dominant, and more rarely, autosomal recessive AI (8–18). Enamelin is a 186 kDa phosphorylated glycoprotein secreted as a precursor into the developing enamel extracellular matrix, where it undergoes a series of proteolytic cleavages (19,20). Its low abundance (1–5% of matrix protein) and localization at the secretory front of the enamel matrix have led to the suggestion that it is important for the regulation of mineral growth during the early stages of enamel formation (21).

Similarly, mutations in the genes encoding matrix metallopeptidase 20 (*MMP20)* and kallikrein related peptidase 4 (*KLK4)* result in autosomal recessive hypomaturation AI (MIM #612529 and #204700) (6,7). Recently, mutations in genes encoding other classes of proteins, distinct from those of the enamel matrix (e.g. *FAM83H*, *WDR72*, *DLX3*, *LAMB3*, *C4orf26* and *SLC24A4*), have also been shown to be associated with AI, underlining the importance of intracellular proteins in enamel biomineralization (22–27).

Recently, we described a mouse manifesting an AI phenotype that was due to a mutation in *Amelx* (*Amelx*^p.Y64H^). We went on to show that affected animals demonstrated chronic activation of the unfolded protein response (UPR). AI in these animals was an example of a proteinopathy giving rise to ER stress, an activated UPR and ultimately ameloblast cell death (28). To test the hypothesis that ER stress and UPR-mediated cell death might be involved in the more frequently observed form of AI caused by ENAM mutations, and so represent a major aetiological mechanism in AI, we have characterized the M100395 mouse model for AI. This mouse harbours a G-T transversion that changes a polar serine residue to a hydrophobic isoleucine at position 55 of the translated enamelin peptide sequence (ENAM^*p.*^^S55I^) (29). Here, we report the detailed characterization of the *Enam^p.^*^S55I^ mouse phenotype and identify a novel heterozygous *ENAM^p.L31R^* mutation in human AI. Comparison of the phenotype of affected enamel of both species strongly suggests that the underlying pathological mechanism is similar and involves ER stress.

## Results

### Incisor teeth of *Enam*^p.S55I^ mice show gross and histological abnormalities

**Figure 1:**
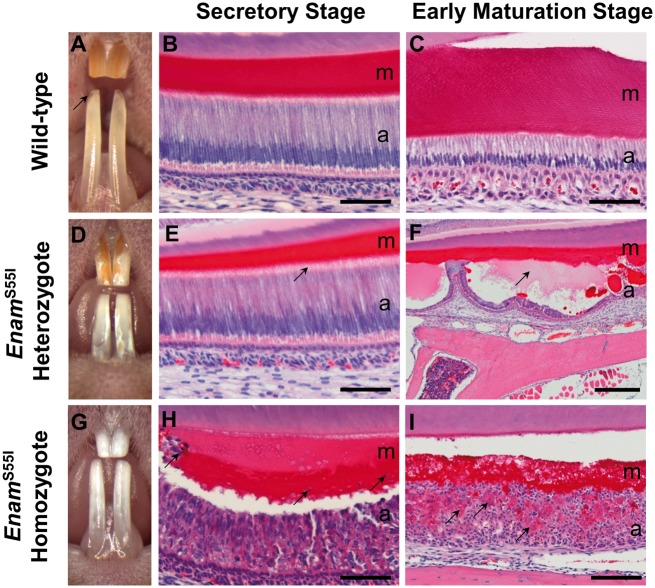
Gross and histological appearance of the incisor teeth of wild-type, *Enam*^S55I^ heterozygous and *Enam*^S55I^ homozygous mice. (**A**) The teeth of wild-type mice have a smooth, orange-coloured, translucent appearance with a sharp incisal edge (arrow). (**B**) Histologically, during the secretory stage of amelogenesis, the enamel organ is composed of tall, columnar ameloblasts which secrete an eosinophilic matrix. (**C**) During the early maturation stage of amelogenesis the ameloblasts have shortened considerably and the matrix has begun to degrade (less intense eosin staining). (**D**) The teeth of *Enam*^S55I^ heterozygous mice are largely white with patches of normal, orange-coloured enamel on their surface. (**E**) The secretory stage ameloblasts (a) are tall and columnar as in wild-type mice but their cytoplasm is more deeply eosin-stained. Contact of the ameloblasts with the matrix is more diffuse (arrow) than in their wild-type counterparts and the secreted matrix is considerably thinner. (**F**) By the early maturation stage, the ameloblasts have frequently lost contact with the matrix forming large blister-like structures (arrow) which contain pale-staining material. (**G**) The teeth of *Enam*^S55I^ homozygous mice are completely white and devoid of enamel. (**H**) The ameloblasts have lost their characteristic columnar structure and form eosinophilic multicellular masses. The matrix produced by these cells is disorganized and contains cellular debris (arrows). (**I**) By the early maturation stage, the ameloblasts continue to form multicellular aggregates enclosing eosinophilic material (arrows) while the matrix appears thin and disorganized. a: ameloblasts; m; matrix. Scale bars: (B, C, E, H) 50 μm; (I), 100 μm; (F), 500 μm.

The enamel of wild-type mouse incisors showed a translucent, orange-coloured appearance with sharp, chisel-like edges at the incisal tip (Fig. 1A). Histologically, the secretory stage enamel organ of the mandibular incisors exhibited ameloblasts with tall columnar morphology surmounting a thick, well-organized eosinophilic enamel extracellular matrix (Fig. 1B). During the maturation stage of amelogenesis, wild-type ameloblasts reduced in height and the enamel extracellular matrix was degraded, as is characteristic of this stage of enamel formation (Fig. 1C).

In *Enam^p.^*^S55I^ heterozygous mice, the incisors were covered by patches of white, opaque, chalky tissue interspersed with material of similar appearance to that seen in the enamel of wild-type mice (Fig. 1D). The secretory stage ameloblasts and secreted enamel matrix were very similar to wild-type animals, although the ameloblast secretory front appeared less well organized and the ameloblast cytoplasm accumulated eosinophilic material compared to wild-type mice (Fig. 1E). From the late secretory/early maturation stages onwards, large numbers of ameloblasts appeared to lose contact with the underlying enamel extracellular matrix, forming large cyst-like structures enclosing pale-staining material (Fig. 1F).

*Enam^p.^* ^S55I^ homozygous mice manifested a more severe phenotype with incisors exhibiting a relatively smooth white surface (Fig. 1G). Differentiated ameloblasts were absent in the secretory stage of amelogenesis; instead, multicellular aggregates were observed below an amorphous eosinophilic matrix (Fig. 1H). As the maturation stage of amelogenesis commenced, the disorganized appearance of the ameloblasts persisted although by now much of the space between them was occupied by matrix-like eosinophilic material (Fig 1I).

### *Enam^p.^* ^S55I^ mice show abnormal expression patterns of enamel matrix proteins

**Figure 2:**
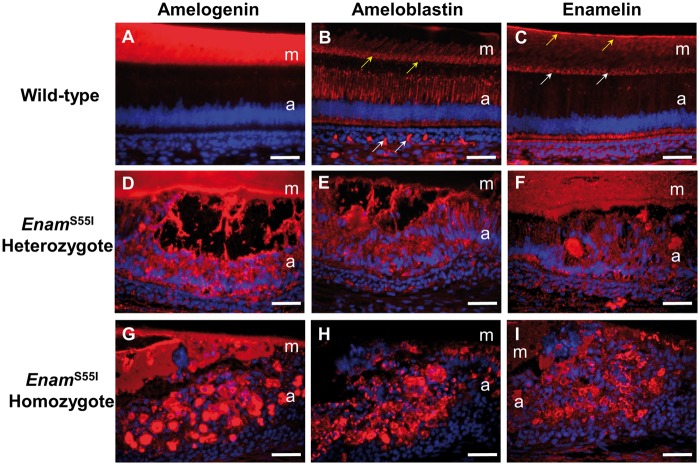
Enamel matrix protein distribution in *Enam*^S55I^ mutant mice. (**A–C**) Wild-type mice. (A) Strong amelogenin immunoreactivity in the extracellular matrix of wild-type mice. (B) Ameloblastin immunoreactivity can be seen within the ameloblasts and at the secretory front (yellow arrows) of the matrix and extending as parallel lines into it. There is non-specific immunolabelling of the vasculature below the ameloblasts (white arrows). (C) Enamelin shows strong immunoreactivity at the dentino-enamel junction (yellow arrows) and at the secretory front (white arrows) with less intense immunostaining throughout the remainder of the matrix. (**D–F**) *Enam*^S55I^ heterozygous mice. (D) In *Enam*^S55I^ heterozygous mice there is strong amelogenin immunoreactivity in the matrix and within the ameloblasts. (E) Little immunoreactivity is observed in the matrix of heterozygous mice but there is strong intracellular immunolabelling for ameloblastin in the ameloblasts. (F) Strong enamelin immunoreactivity is seen throughout the matrix and within the ameloblasts. (**G–I**) *Enam*^S55I^ homozygous mice. (G) There is intense amelogenin immunoreactivity both in the matrix and within the ameloblasts. (H) Strong ameloblastin immunolabelling is confined to the ameloblasts. (I) There is strong enamelin immunoreactivity within the ameloblasts and weaker immunostaining of the matrix. a: ameloblasts; m; matrix. Scale bars: 50 μm.

Immunolabelling of the enamel matrix proteins amelogenin, ameloblastin and enamelin in sections of wild-type secretory enamel organ showed a typical distribution, with amelogenin occupying the whole of the secreted enamel matrix while ameloblastin and enamelin were present predominantly at the secretory front of the enamel matrix and at the dentino-enamel junction (Fig. 2A–C). Additionally, as is usually seen in wild-type secretory ameloblasts, ameloblastin expression was also detected within the ameloblasts in a supranuclear position (Fig. 2B). Ameloblasts of *Enam^p.^*^S55I^ heterozygous mice retained amelogenin, ameloblastin and enamelin within their cytoplasm, while the ‘blister-like’ structures formed by these ameloblasts also exhibited strong immunoreactivity for each protein (Fig. 2D–F). The distribution of enamel matrix proteins was also abnormal in mice homozygous for the *Enam^p.^*^S55I^ mutation. Here, the ameloblasts were rounded and often contained all three enamel matrix proteins within their cytoplasm (Fig. 2G–I). In the immediate extracellular space, the distribution of amelogenin and enamelin were highly disorganized while ameloblastin was undetectable. The enamel matrix that was present was immunoreactive for amelogenin and enamelin (Fig. 2G and I).

### Keratin 14 immunolabelling shows abnormalities at the secretory front in *Enam^p.^*^S55I^ heterozygous mice

Using keratin 14 immunolabelling, the terminal web region of the secretory ameloblasts from which the specialized secretory structures, the Tomes’ processes, project into the enamel matrix was readily observed in wild-type mice (Supplementary Material, Fig. S1A). This region and its associated Tomes’ processes were abnormal at the early stages of enamel matrix secretion in *Enam^p.^*^S55I^ heterozygous mice; the terminal web region appearing more diffuse and the Tomes’ processes more disorganized than in wild-type animals (Supplementary Material, Fig. S1B). In the later stages of enamel matrix secretion, ameloblasts in *Enam^p.^*^S55I^ heterozygous mice lost contact with the underlying matrix following retraction of the Tomes’ process (Supplementary Material, Fig. S1C). The ameloblast phenotype in *Enam^p.^*^S55I^ homozygous mice was so severe that it was impossible to make meaningful comparisons with wild-type or heterozygous mice.

### Ameloblasts in *Enam^p.^*^S55I^ mice show ultrastructural abnormalities

**Figure 3:**
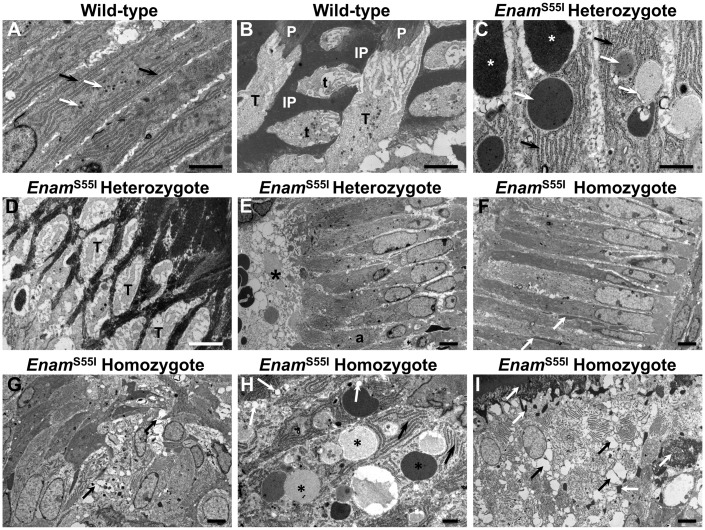
Ultrastructure of *Enam*^S55I^ ameloblasts. (**A, B**) Wild-type secretory stage ameloblasts (A) display a tall columnar morphology with abundant, peripherally organized rough endoplasmic reticulum (black arrows) surrounding a centrally placed Golgi apparatus and associated small vesicles (white arrows). (B) At the ameloblast tip is a specialized secretory structure, the Tomes’ process (T) that secretes enamel matrix in such a manner that the matrix that is deposited from the tip gives rise to enamel prisms (P) while that derived from its flanks produces inter-prismatic (IP) enamel. Adjacent Tomes’ processes, sectioned more transversely, can also be seen and these secrete their enamel matrix in a different orientation, (t), to give rise to the interlocking arrangement of enamel prisms. **(C–E)***Enam*^S55I^ heterozygous mice the secretory ameloblasts also have (C) abundant rough endoplasmic reticulum (black arrows) but also contain larger intracellular vesicles of varying electron translucency (white arrows) when compared to wild-type. Extracellular accumulation of matrix-like material is also seen just under the secretory front of the ameloblasts (white asterisks). (D) The Tomes’ processes of these ameloblasts are abnormal (T) appearing disorganized, crowded and slim. (E) As the maturation stage of amelogenesis is approached, the ameloblasts (a) of *Enam*^S55I^ heterozygous mice retract their Tomes’ processes and lose contact with the enamel matrix and form cyst-like structures (black asterisk) containing matrix-like material. (**F–I**) The secretory stage ameloblasts of *Enam*^S55I^ homozygous mice are initially columnar (F) with accumulations of matrix-like material between them (white arrows). (G) Later in the secretory stage of amelogenesis, *Enam*^S55I^ homozygous ameloblasts lose their columnar morphology and become ovoid or spherical. Many of the cells contain large vacuoles (black arrows). (H) At higher magnification the ameloblasts of *Enam*^S55I^ homozygous mice contain prominent rough endoplasmic reticulum (black arrows). Large vesicles containing material of strong to weak electron translucency (black asterisks) as well as vacuolar structures (white arrows) are often seen in these ameloblasts. (I) At later stages of amelogenesis, homozygous ameloblasts show extensive vacuolation (black arrows) and accumulations of matrix like material appear around and between them (white arrows). Scale bars: (A, B, C, D), 2 μm; (E, F, G), (I) 5 μm; (H) 1 μm.

At the ultrastructural level, wild-type secretory stage ameloblasts exhibited a tall columnar epithelial morphology containing prominent rough endoplasmic reticulum (RER), central Golgi apparatus and small cytoplasmic vesicles (Fig. 3A). Tomes’ processes were present apically, interdigitating with the newly formed enamel matrix (Fig. 3B). In *Enam^p.^*^S55I^ heterozygous mice, the secretory stage ameloblasts also showed a columnar epithelial morphology, prominent RER, central Golgi apparatus and cytoplasmic vesicles (Fig. 3C). However, the vesicles were often considerably larger than those observed in wild-type mice (Fig. 3C). Notably, the Tomes’ processes of the ameloblasts of *Enam^p.^*^S55I^ heterozygous mice were disorganized, crowded and exhibited a spindle-like appearance compared to those of wild-type mice (Fig. 3D). Eventually, the *Enam^p.^*^S55I^ heterozygous ameloblasts lost contact with the enamel matrix as the secretory stage of amelogenesis progressed and formed cyst-like structures containing electron translucent material (Fig. 3E). Electron dense, matrix-like material was often seen between these ameloblasts (Fig. 3E).

The pre-secretory stage ameloblasts of *Enam^p.^*^S55I^ homozygous mice initially assumed a columnar morphology but failed to develop a Tomes’ process (Fig. 3F). Instead, these cells lost their columnar morphology, adopted an ovoid/spherical appearance and formed irregular cellular masses (Fig. 3G). These cells were frequently vacuolated (Fig. 3G) and the cytoplasm filled progressively with large vesicular structures containing electron translucent to electron dense material consistent with the retention of enamel matrix proteins (Fig. 3H). Eventually, the abnormal ameloblasts degenerated, leaving much cellular debris and deposits of enamel matrix-like material (Fig. 3I).

### Enamel architecture of *Enam^p.^*^S55I^ mice is abnormal and disorganized

**Figure 4:**
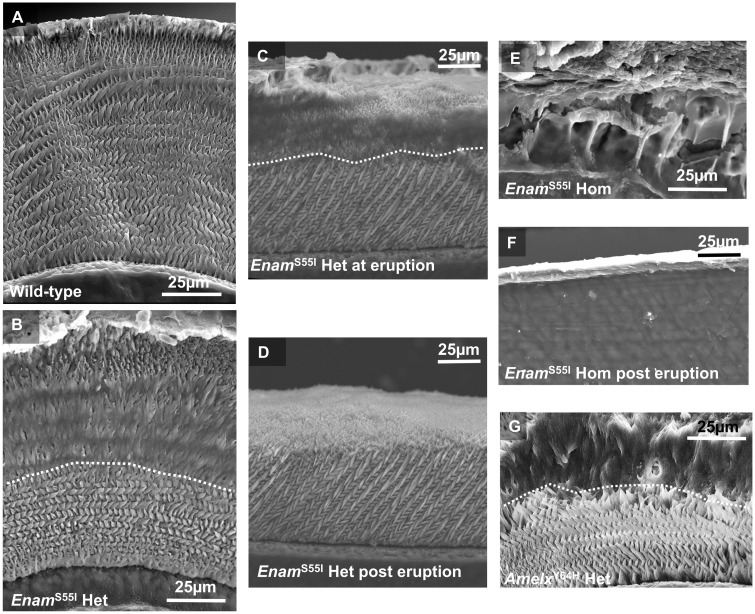
** **SEM comparing the structure of wild-type, heterozygous and homozygous *Enam*^S55I^ and female heterozygous *Amelx^Y64H^* mandibular incisor enamel. **(A)** Transverse sections through a wild-type incisor at eruption show the well organized, decussating pattern of enamel prisms characteristic of rodent incisor enamel. Only the very outermost layer of enamel is aprismatic. **(B)** In contrast, heterozygous *Enam*^S55I^ enamel exhibits a structurally normal decussating inner layer of enamel topped with a subsequently secreted layer that is structurally abnormal. A dotted white line marks the boundary between the two layers**. (C)** Sagittal sections through heterozygous *Enam*^S55I^ enamel at the point of eruption show that a structurally abnormal outer enamel layer is present. **(D)** Later, in post-eruptive enamel nearer the incisal biting edge, the abnormal layer has been lost and only the inner structurally normal enamel layer survives. **(E)** Homozygous *Enam*^S55I^ mice fail to secrete any tissue with structural resemblance to normal enamel (unerupted transverse section). **(F)** Post-eruptive homozygous *Enam^pS55I^* incisors exhibit no enamel layer; just a thin layer of material of unknown composition. (**G)** The phenotype of heterozygous *Amelx^pY64H^* mouse enamel (39) is remarkably similar to that of heterozygous *Enam^pS55I^ mice*; both exhibiting a structurally normal inner layer and an abnormal outer layer.

Scanning electron microscopy **(**SEM) of unerupted wild-type incisors showed the typical decussating arrangement of enamel prisms interspersed with aprismatic material that is characteristic of rodent incisor enamel (Fig. 4A). In contrast, unerupted mandibular incisor enamel from *Enam^p.^*^S55I^ heterozygous mice was divided into two architecturally-distinct regions (Fig. 4B). The inner region, corresponding to tissue initially secreted by the ameloblasts, comprised decussating enamel prisms similar to wild-type enamel. In contrast, an outer region, corresponding to enamel that had been secreted by the ameloblasts at a later time, consisted of material in which the normal decussating prism architecture was highly disturbed. This disturbed outer enamel layer was present in newly erupted tissue (Fig. 4C) but was quickly lost, presumably due to masticatory wear (Fig. 4D). In contrast, the apparently normal inner layer of prismatic enamel, corresponding to that initially secreted by ameloblasts, was more resilient, and survived to some extent in the oral environment. (Fig. 4D). Mandibular incisors from *Enam^p.^*^S55I^ homozygous mice failed to exhibit any obvious enamel layer in either unerupted or erupted tissue (Fig. 4E and F, respectively).

We noted the striking similarity between the enamel phenotype of unerupted *Enam^p.^*^S55I^ heterozygous mice (Fig. 4B) and the phenotype of incisor enamel from female mice heterozygous for the *Amelx*^p.Y64H^ mutation that we reported previously (Fig. 4G).

### Enamel mineral quality and quantity is compromised in *Enam^p.^*^S55I^ mice

**Figure 5:**
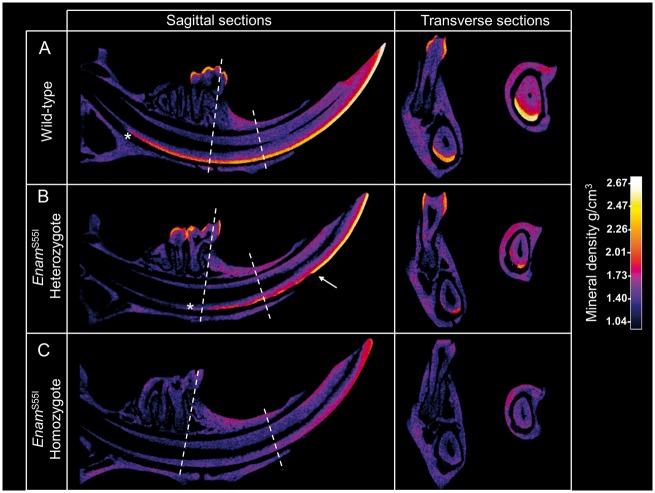
Calibrated micro X-ray computed tomography comparing mineral density in wild-type, heterozygous and homozygous *Enam*^S55I^ enamel. **(A)** Sagittal CT sections show that wild-type incisor enamel becomes X-ray opaque due to increased mineral density (secondary mineralization) that occurs at the beginning of the maturation stage. Mineral density increases gradually throughout maturation and following eruption. Transverse CT sections, taken at points indicated by the dotted lines, show the enamel layer is complete across the width of the labial aspect of the incisor. **(B)** In contrast, onset of secondary mineralization is delayed in heterozygous *Enam*^S55I^ enamel (asterisk) and only approaches the density seen in wild-type mice following eruption. Transverse sections show that secondary mineralization is compromised across the whole width of the enamel**. (C)** Homozygous *Enam*^S55I^ do not exhibit any X-ray opaque material corresponding to incisor enamel. Only wild-type and heterozygous *Enam*^S55I^ mice exhibit molar enamel (A and B). The molar teeth of all mice are fully erupted in these mice and no developing enamel is present.

Micro X-ray computed tomography (µCT) scans of wild-type and *Enam^p.^*^S55I^ mandibular incisors were taken through sagittal and transverse planes and calibrated to show quantitative mineral density (Fig. 5). As expected, wild-type incisors were characterized by a covering of enamel on the labial aspect of the incisor (Fig. 5A) that became X-ray opaque as the maturation stage of amelogenesis advanced and enamel mineral density increased. The enamel density continued to increase following eruption due to the presence of mineral ions in the saliva (post-eruptive maturation). The molar teeth, which do not continually develop, and are therefore present in their completed form, can also be seen, exhibiting typical fully mature erupted enamel.

Incisors from *Enam^p.^*^S55I^ heterozygous mice differed from wild-type in that the developing enamel matrix became X-ray opaque more anteriorly, indicating a delay in the onset of maturation stage secondary mineralization (Fig. 5B). Thereafter, enamel mineralization was irregular. However, on eruption, the enamel appeared to undergo considerable post-eruptive mineralization such that the surviving enamel was more uniformly mineralized and to a higher density (arrowed) than the unerupted tissue. In some cases, the boundary between the structurally normal enamel and the abnormal outer enamel described previously was visible as a hypomineralized region (Supplementary Material, Fig. S2). Mature molars from *Enam^p.^*^S55I^ heterozygous mice also appeared to be mineralized, though we are unable to attribute this to post-eruptive mineralization as these teeth were already fully developed and erupted in these animals.

In contrast to the *Enam^p.^*^S55I^ heterozygous mice, incisors and molars from *Enam^p.^*^S55I^ homozygotes were devoid of any X-ray opaque tissue where enamel would normally be present (Fig. 5C).

An animated supplemental video is provided online showing rendered reconstructions of mandibles from heterozygous *Enam^p.^*^S55I^ and wild-type mice. This video further illustrates the structural and compositional defects in both erupted and unerupted incisor enamel associated with the *Enam^p.^*^S55I^ mutation.

### *Enam^p.^* ^S55I^ ameloblasts show evidence of endoplasmic reticulum stress

**Figure 6:**
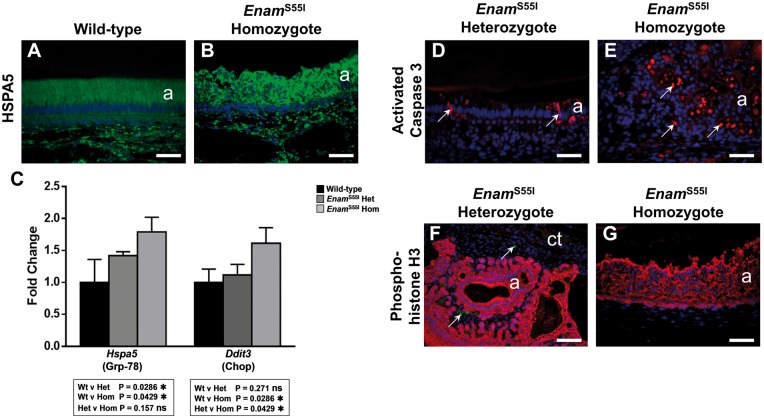
Analysis of cell death, proliferation and ER stress in *Enam*^S55I^ mutant mice. (**A, B**) Immunolabelling for the ER chaperone HSPA5 (Grp-78) showed moderate immunoreactivity in wild-type secretory stage ameloblasts (A) and also in those of *Enam*^S55I^ heterozygous mice (data not shown). The ameloblasts of *Enam*^S55I^ homozygous mice (B) showed a substantial increase in HSPA5 immunofluorescence. **(C)** Quantitative reverse transcriptase PCR analysis of ER stress gene expression in the secretory stage enamel organ. *Hspa5* (Grp-78) gene expression was statistically significantly elevated in *Enam*^S55I^ heterozygous and homozygous mice compared to wild-type. *Ddit3* (Chop) gene expression, was statistically significantly elevated in *Enam*^S55I^ homozygous mice but not in *Enam*^S55I^ heterozygous secretory stage enamel organ compared to wild-type. The data was analysed using the one-sided Mann-Whitney test. **(D, E)** Immunolabelling for activated caspase 3 in *Enam*^S55I^ heterozygous ameloblasts (D) shows occasional apoptotic ameloblasts at the transition stage of amelogenesis (arrows) as is typically seen in wild-type animals (data not shown). In *Enam*^S55I^ homozygous mice, many of the aberrant ameloblasts in the multicellular masses were seen to be undergoing apoptosis (arrows) (E). (**F, G**) Dual immunolabelling for phosphohistone H3 (green fluorescence), a marker of cell proliferation and keratin 14 (red fluorescence), to discriminate the enamel organ from the surrounding connective tissue, showed that in all mutant mice studied none of the ameloblast population showed evidence of proliferation. (F) Where proliferation was observed, this was confined to the surrounding connective tissue fibroblast population (arrows). Scale bars: (A, B, D, E, G), 50 μm; (F), 100 μm.

Expression of mutant proteins can lead to the retention and accumulation of misfolded protein intermediates within the endoplasmic reticulum (ER), triggering the UPR (30). Increased expression of the ER luminal chaperone HSPA5 (GRP-78, BiP) is central to this process as the cell attempts to restore the protein folding capacity of the ER. Immunohistochemical detection of HSPA5 in wild-type incisors (Fig. 6A) revealed moderate immunoreactivity within the secretory ameloblasts consistent with their strong protein synthetic activity during this stage of amelogenesis. By contrast, in secretory ameloblasts of *Enam^p.^*^S55I^ homozygous mice, HSPA5 immunofluorescence was substantially greater than that seen in wild-type animals (Fig. 6B). Quantitative RT-PCR (RTqPCR) analysis of cDNA derived from micro-dissected, secretory-stage ameloblasts of wild-type and *Enam^p.^*^S55I^ mice for *Hspa5* gene expression levels showed statistically significant upregulation in both heterozygous (*P* = 0.0286) and homozygous (*P* = 0.0429) mice compared to wild-type (Fig. 6C).

*Ddit3* (*Chop*) encodes a protein involved in triggering apoptotic cell death during periods of prolonged ER stress. RTqPCR analysis of cDNA from the secretory ameloblasts of *Enam^p.^*^S55I^ heterozygous mice showed that *Ddit3* expression was not significantly different to wild-type whereas expression in *Enam^p.^*^S55I^ homozygous mice was increased significantly compared to both wild-type (*P* = 0.0286) and *Enam^p.^*^S55I^ heterozygous (*P* = 0.0429) mice (Fig. 6C).

### Ameloblasts in *Enam^p.^*^S55I^ homozygous mice show increased apoptosis but not proliferation

Apoptosis is a normal feature of amelogenesis with approximately 50% of the ameloblast population being culled during the transition and maturation stages.

Immunohistochemistry using an antibody to activated caspase-3, a key effector of apoptosis, in incisors from *Enam^p.^*^S55I^ heterozygous mice revealed occasional ameloblasts with cytoplasmic immunoreactivity towards the end of the secretory stage of amelogenesis (Fig. 6D), an appearance that is indistinguishable from wild-type mice (data not shown). Activated caspase-3 immunolabelling of incisors from *Enam^p.^*^S55I^ homozygotes showed large numbers of caspase-3-positive presumably apoptotic ameloblasts, in the multicellular masses that arise at the secretory stage in these mice (Fig. 6E).

Caspase-3 is also associated with cellular processes outside apoptosis such as proliferation (31). To test whether or not the abnormal caspase-3 distribution detected in the ameloblasts of *Enam^p.^*^S55I^ homozygous mice was associated with dysregulated ameloblast proliferation, we used antibodies to phospho-histone H3 to identify any abnormal cell proliferation in the secretory stage enamel organ distal to the proliferative base of the incisor. Immunolabelling for keratin 14 was also carried out in these preparations to discriminate cells of the enamel organ from those of the surrounding connective tissue. No enamel organ cells distal to the proliferative base were immunoreactive with the phospho-histone H3 antibody in any of the specimens examined (Fig. 6F and G) although some cells were immunolabelled in the surrounding connective tissue (e.g. Fig. 6F) in *Enam*^S55I^ heterozygous mice. At the apices of the mandibular incisors, where new ameloblasts are continuously generated from a stem cell pool, phosphohistone H3 positive cells were observed in all mice examined (data not shown).

### A novel *ENAM*^p.L31R^ mutation causes AI in humans

**Figure 7:**
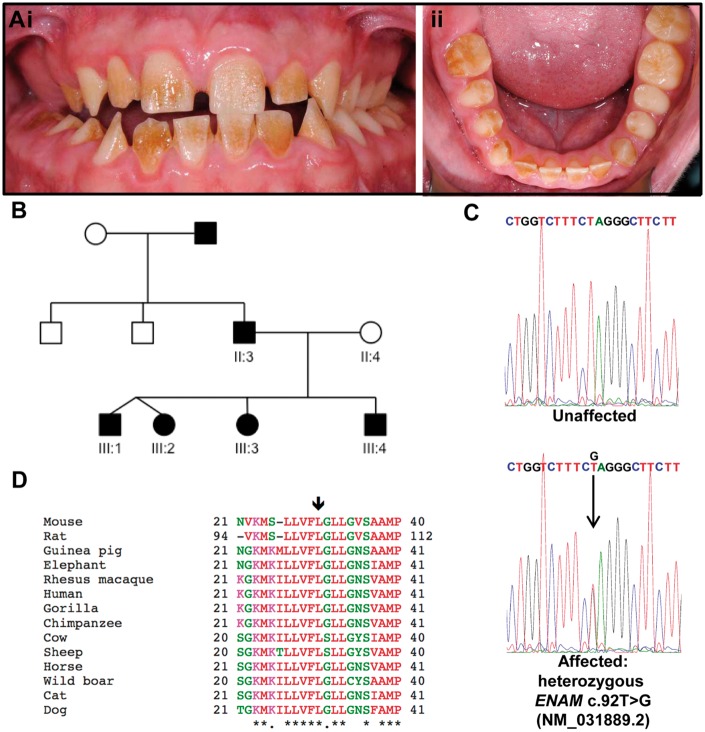
(**Ai and ii**) The permanent dentition of III:4 is characterized by a generalized rough hypoplastic AI with superficial exogenous staining. **(B)** Pedigree of family 1. Individuals are not necessarily shown in age order. Affected individuals are indicated by shading. DNA was available from labelled individuals. Whole exome sequencing was carried out using DNA from III:2. Teeth were obtained from III:1 and III:4. **(C)** Sanger sequencing of *ENAM* exon 3 confirmed the heterozygous c.92T > G variant (NM_031889.2), originally identified in III:2 by WES, segregated with the disease phenotype in all available family members. **(D)** Clustal Omega multiple sequence alignment of homologous ENAM protein sequences. The arrow indicates the residue altered by the c.92T > G variant, p.L31R (NM_031889.2; NP_114095.2). The affected residue and the ten residues that flank it to either side are included in the alignment. ENAM sequences used: Mouse, *Mus musculus* NP_059496.1; Rat *Rattus norvegicus* NP_001099471.1; Guinea pig, *Cavia porcellus* XP_003467585.2; Elephant, *Loxodonta africana* XP_003414215.1; Rhesus macaque, *Macaca mulatta* XP_014994062.1; Human, *Homo sapiens* NP_114095.2; Gorilla, *Gorilla gorilla* XP_004038829.1; Chimpanzee, *Pan troglodytes* XP_526591.1; Cow, *Bos taurus* XP_605463.4; Sheep *Ovis aries* XP_004009936.1; Horse *Equus caballus* XP_001487944.1; Wild boar, *Sus scrofa* NP_999406.1; Cat, *Felis Catus* XP_003985351.1; Dog *Canis lupus familiaris* XP_539305.3.

We identified a white British family segregating autosomal dominant hypoplastic AI (Fig. 7A and B). Whole exome sequencing (WES) of individual III:2 was performed and a heterozygous variant in enamelin (*ENAM*; OMIM *606585), c.92T > G, p.(L31R) (NM_031889.2; NP_114095.2) identified. Sanger sequencing confirmed the variant and demonstrated that it segregated with the disease phenotype in all available members of the family (Fig. 7C). The c.92T > G p.L31R variant is predicted to be pathogenic by a variety of mutation pathogenicity prediction software (Supplementary Material, Table S1) and was absent from publically available databases of variation, including dbSNP146 (32), Exome Variant Server (http://evs.gs.washington.edu/EVS/) and Exome Aggregation Consortium (v0.3; http://exac.broadinstitute.org/). WES and Sanger sequencing of DNA from an additional four white British families segregating autosomal dominant hypoplastic AI showed that the same variant was present in all four families and segregated with the disease phenotype in all cases (Supplementary Material, Fig. S3). Investigation of orthologous protein sequences (Fig. 7D) found that the L31 residue is conserved in all species analysed and resides within a conserved hydrophobic region within the ENAM signal peptide.

### Enamel architecture associated with *ENAM^p.^*^L31R^ in human AI is abnormal

**Figure 8:**
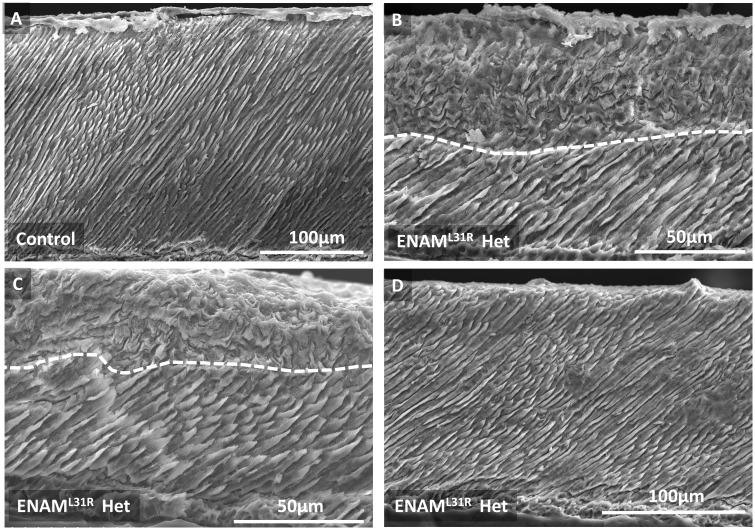
SEM comparison between human control primary incisor enamel and primary incisor enamel suffering the effects of the heterozygous ENAM^pL31R^ mutation. **(A)** Control enamel exhibits the typical prismatic structure with organized prisms spanning the entire enamel thickness**. (B, C)** In contrast, affected enamel recapitulates the phenotype seen in heterozygous Enam^pS55I^ and Amelx^pY64H^ mice in that it exhibits an inner layer of enamel exhibiting ordered prismatic structure overlaid with an outer enamel layer that is structurally abnormal (the two layers being demarcated by the white dotted line). **(D)** In some areas, affected enamel appears structurally normal and does not exhibit a structurally abnormal outer layer**.** However, it is not clear whether these apparently unaffected areas simply represent areas where the structurally abnormal outer layer has been lost due to wear and tear experienced in the mouth.

SEM of a primary incisor from an individual heterozygous for an *ENAM^p.^*^L31R^ mutation showed the presence of a structurally normal inner layer of enamel, around 50 µm in thickness, with a clearly demarcated structurally abnormal outer layer of varying thickness (Fig. 8). The presence of a normal inner enamel layer and an abnormal outer layer is strikingly similar to the phenotype observed in *Enam^p.^*^S55I^ mice (Fig. 4). Enamel present on the upper crown exhibited a layer of enamel that was in general structurally normal in appearance though at 50–100 µm in thickness considerably thinner than enamel on an unaffected control primary incisor. We hypothesize that an abnormal enamel layer was present on the upper crown but since this area of the tooth would have experienced the greatest amount of attrition in the mouth the abnormal layer has been lost leaving behind the structurally normal inner layer that survives in the mouth.

## Discussion

Dominant mutations in the *ENAM* gene are frequently associated with AI in humans (8,10–18). Our results are consistent with this observation as both heterozygous and homozygous *Enam^p.^*^S55I^ mice manifest a disturbed enamel phenotype. The murine data also support observations in human examples of *ENAM*-linked AI where the disease-causing allele shows a dosage effect (9,16). The validity of the mouse model is further reinforced by the remarkable similarity between the heterozygous *Enam^p.^*^S55I^ mouse enamel phenotype and the phenotype exhibited in the case of human AI presented here caused by a heterozygous *ENAM*^p.L31R^ mutation (Figs. 4 and 8).

One aim of the current work was to test the hypothesis that AI in *Enam^p.^*^S55I^ mice is a proteinopathic disease driven by ER stress and an activated UPR that affects ameloblast function ultimately leading to ameloblast apoptosis. In addition, we wished to test the hypothesis that proteinopathy may be a factor in human AI. However, it is not feasible to study amelogenesis in humans at the molecular level; instead our approach was to study exfoliated human teeth and compare the enamel phenotype to phenotypes observed in well-characterized mouse AI models where we believe the phenotype is diagnostic for the disease aetiology. To this end, we have identified a case of human AI linked to an *ENAM^p.L31R^* mutation for which exfoliated teeth were available for study.

Proteinopathies are generally related to the misfolding of proteins in the secretory pathway. Misfolding may be due to a mutation in the protein itself or to compromised function of ancillary proteins (e.g. chaperones, kinases etc.) responsible for folding, post-translational modification and trafficking of the nascent translation product (33). Misfolded proteins, exhibiting inappropriate protein-protein interactions and aggregation in the secretory pathway, are increasingly recognized as being responsible for a wide range of ‘conformational’ diseases including, Alzheimer’s disease, Huntington’s disease, atherosclerosis, cystic fibrosis and type 2 diabetes (34,35). When misfolded proteins accumulate, membrane sensors (IRE1α, PERK and ATF6) in the ER membrane trigger the UPR in an attempt to relieve ER stress and restore homeostasis (36). The UPR is a complex homeostatic network of signalling pathways that has a varied range of effects on cell function depending on the precise nature of the challenge. The initial UPR response to ER stress attempts to relieve the stress via multiple mechanisms, including reducing the general secretory load, increasing the volume of the ER, and upregulating the synthesis of chaperones such as HSPA5 to increase rescue of protein misfolding. In addition, the UPR can directly modulate a number of metabolic processes such as energy metabolism and lipid synthesis. The signalling outputs of the UPR are graded and mirror the intensity of the ER stress (37) but ultimately, if homeostasis cannot be restored, the UPR switches from pro-survival mode into a pro-apoptotic mode including upregulation of pro-apoptotic transcription factors such as DDIT3/CHOP (38).

We previously showed that ameloblasts of mice carrying the *Amelx*^p.Y64H^ mutation retained amelogenin in multiple intracellular vesicles triggering persistent ER stress that eventually induced cell death mediated via the UPR (39). In the current investigation, we have observed a very similar aetiology in *Enam^p.^*^S55I^ mice. In *Enam^p.^*^S55I^ mice, the ordered relationship between the ameloblast monolayer and the enamel matrix being secreted is disturbed, enamel protein secretion is impaired, ER stress is increased, and the UPR is activated.

The similarities between *Amelx*^p.Y64H^ and *Enam^p.^*^S55I^ mouse enamel extend to the characteristic structural enamel phenotype observed, suggesting that this phenotype may be characteristic for AI where ER stress is involved. Mice heterozygous for both *Amelx*^p.Y64H^ and *Enam^p.^*^S55I^ secrete an initial layer of enamel that appears structurally normal in terms of its prismatic structure but enamel secreted subsequently is structurally abnormal and disorganized in both genotypes (Fig. 4B and G). This abnormal outer layer is quickly lost following eruption, presumably by attrition, while the structurally ‘normal’ inner enamel layer survives to some extent suggesting retention of functionality (compare Fig. 4C and D). The reason why the abnormal outer layer is lost on tooth eruption may be related to a hypomineralized boundary seen using µCT between the two distinct layers (Supplementary Material, Fig. S2) representing a ‘fault line’ susceptible to the shear forces encountered during mastication.

Clearly, any proposed aetiological mechanism operating in the *Enam^p.^*^S55I^ heterozygous mice needs to account for the enamel phenotype described above. The fact that approximately 50 µm of structurally normal enamel is secreted initially indicates that *Enam^p.^*^S55I^ translation products have little or no impact on the ability of ameloblasts to produce the characteristic decussating enamel prism structure seen in rodents. Presumably, sufficient wild-type enamelin is produced and secreted to fulfil the functional role of enamelin. However, thereafter, ameloblast function is perturbed leading to the production of structurally abnormal enamel.

Simple eosin staining in the cytoplasm of secretory ameloblasts of affected mice (Fig. 1) together with the immunohistochemical detection of intracellular retention of the enamel extracellular matrix proteins amelogenin, ameloblastin and enamelin (Fig. 2) indicated an impairment of the ameloblast secretory pathway. TEM data showed that affected ameloblasts frequently contained large intracellular vesicles consistent with enamel protein retention (Fig. 3).

ENAM is autosomal in mice and humans and assuming bi-allelic expression, all of the ameloblasts will be affected by the *Enam^p.^*^S55I^ mutation. However, homozygous mice are more severely affected as both copies of *Enam* are mutated which effectively doubles the concentration of mutated ENAM^p.S55I^ entering the ER when compared to heterozygotes. This could increase the ER stress generated during intracellular trafficking leading to a more draconian UPR and an apoptotic end point. In support of this, expression of the pro-apoptotic transcription factor DDIT3 was significantly increased in enamel organ cells of *Enam^p.^*^S55I^ homozygous mice compared to both wild-type and heterozygous litter-mates. In contrast, enamel organ cells of *Enam^p.^*^S55I^ heterozygous mice do not appear to suffer increased rates of apoptosis as expression of *Ddit3* was not significantly different from wild-type (Fig. 6C). Although ameloblasts from *Enam^p.^*^S55I^ heterozygous mice do not suffer increased levels of *Ddit3* expression (apoptosis), the intracellular retention of proteins (Figs. 1–3) still causes a significant upregulation of *Hspa5* (Fig. 6C) indicative of ER stress and an activated UPR (40). Taken together, the data suggest that ameloblasts of *Enam^p.^*^S55I^ heterozygous mice experience ER stress and an activated UPR. We suggest that the UPR, acting in pro-survival mode, permits these cells to secrete an initial structurally normal layer of enamel despite indications that the Tomes’ processes may be thinner than wild-type (Fig. 3). Presumably, enough wild-type enamelin from the unaffected allele in each ameloblast is produced to carry out its normal extracellular function (which itself remains unclear). However, as the ER stress becomes chronic, the UPR signal output undergoes subtle modification; not signalling a full-blown apoptotic event, but rather, a less dramatic response such as further limiting protein translation in an attempt to relieve the ER stress. Even though the UPR does not switch to full apoptotic mode, the observed phenotype indicates that heterozygous ameloblast function is further compromised at this point and the ameloblasts lose the ability to produce structurally normal prismatic enamel and instead produce the structurally and functionally abnormal enamel compromising the outer enamel layer.

We might speculate that whatever impact the UPR is having on ameloblast function, it may manifest itself phenotypically via an effect on the Tomes process as the Tomes process is widely regarded as essential to the production of prismatic enamel.

µCT scanning showed that the onset of secondary mineralization of the enamel matrix was delayed in *Enam^p.^*^S55I^ heterozygous mice compared to wild-type (Fig. 5A and B). Under the influence of maturation stage secondary mineralization, unerupted wild-type enamel gradually increased in mineral density from a density of around 2.0 g/cm^3^ to around 2.3 g/cm^3^ at the point of eruption. In contrast, the unerupted enamel of *Enam^p.^*^S55I^ heterozygotes was poorly mineralized. Much of the tissue had a similar density to the underlying dentine (∼1.5 g/cm^3^) with sporadic patches of enamel exhibiting a higher density nearer to 2.0 g/cm^3^. We suggest that affected ameloblasts were so compromised due to the effects of ER stress and general disorganization that one of their normal maturation stage functions, which is to actively pump mineral ions into the enamel matrix to drive secondary mineralization (3), was compromised.

However, the enamel matrix clearly retained the *capacity* to mineralize as mineral density increased dramatically post- eruption when the enamel was exposed to saliva and the structurally normal inner enamel layer appeared to resist masticatory wear (Fig. 5B arrowed). In contrast, the outer structurally abnormal outer layer, was quickly lost presumably due to its inability to withstand masticatory forces (Fig. 4D).

Molars in the *Enam^p.^*^S55I^ homozygous mice, like the incisors, were completely lacking enamel. However, molars in heterozygous mice appeared similar to wild-type molars in terms of mineral density. We hypothesize that any delay in maturation stage mineralization, similar to that observed in the heterozygous incisors, would be compensated for by post-eruptive mineralization that occurs in mouse molars once they are in contact with saliva (41).

µCT confirmed that the incisors (and molars) of *Enam^p.^*^S55I^ homozygous mice failed to produce any normal enamel layer emphasizing the critical role played by enamelin in amelogenesis and/or the toxicity ensuing when 100% of the enamelin produced is mutated.

The effect of the *Enam^p.^*^S55I^ mutation on the biochemical properties of the translated protein and any resulting loss of function or gain of toxicity is unclear. However, the replacement of the relatively small polar amino acid serine with the larger non-polar hydrophobic amino acid isoleucine is likely to be significant. The alkyl R group of isoleucine limits the conformational options for the polypeptide chain during folding in the ER (42). The effect of the point mutation may therefore be to cause misfolding, increased protein-protein interactions and subsequent ER stress. S55 is also a potential phosphorylation target of the kinase Fam20c (43). If S55 is indeed phosphorylated, the substitution of serine carrying a charged hydophilic phosphate group for isoleucine would change the biochemical character of this site even more dramatically.

Although almost impossible to prove without access to affected developing human AI teeth, we believe our data provides evidence that the novel heterozygous *ENAM^p.L31R^* human mutation reported here may also drive ER stress. The heterozygous human *ENAM^p.L31R^* phenotype closely mimics the highly characteristic phenotype seen in heterozygous *Enam^p.^*^S55I^ mice where the enamel layer is bifurcated into a structurally normal inner layer and a structurally abnormal outer layer. The similar phenotypes suggest that AI linked to the heterozygous *ENAM^p.L31R^* mutation shares a similar disease mechanism based on an evolving UPR that perturbs ameloblast function at a specific time point resulting in a switch from producing structurally normal enamel to producing structurally abnormal enamel. Sequence analysis of wild-type *ENAM* using the Phobius prediction tool (44) places residue L31 in the hydrophobic core region (H-region comprising residues 25–33) of the 39 residue *ENAM* signal sequence. The H-region is a critical component in the targeting of secretory proteins. Once the signal sequence emerges from the ribosome, the H-region interacts with the signal recognition particle and translation is paused while the complex delivered to the ER membrane where the ribosome and nascent peptide are transferred to the translocon. Translation resumes and the growing peptide is translocated into the ER lumen. Once translation is complete the signal sequence is usually cleaved and the protein is released. Mutations in the H region can abolish translocation and the correct cleavage of the signal peptide. In the presence of the L31R mutation Phobius no longer predicted the presence of a signal peptide. The actual impact of the *ENAM^p.L31R^* mutation on enamelin translocation and secretion needs to be verified experimentally. However, highly relevant to the *ENAM^p.L31R^* mutation, is a previously published report detailing a C18R substitution in the H-region of the signal sequence in human preproparathyroid hormone. This mutation still permitted parathyroid hormone to be translocated into the ER but subsequently the protein became trapped in the ER triggering ER stress and classic UPR induced apoptosis (45).

In summary, our data indicate that cellular stress provoked by a failure of mutant enamelin to adopt the correct conformation within the ER is an aetiological driver leading to AI in *Enam^p.^*^S55I^ mice. This mutation is therefore another example of AI as a conformational disease. A number of pharmacological agents are available to overcome the effects of ER stress acting in various ways such as increasing folding protein folding capacity or inhibiting apoptosis (46). We have already used one such compound, 4-phenybutyrate, in *Amelx*^p.Y64H^ heterozygous female mice, to rescue the AI phenotype (39). The *Enam*^S55I^ mouse therefore will provide a further opportunity for us to test the efficacy of such compounds as potential treatments for AI and provide information on the wider applicability of such compounds for treating proteopathic disease in general. Such information could have clinical value, since the human case of AI reported here linked to a mutation in the H-region of the enamelin signal sequence may be based on a similar ER stress-linked aetiology. It is noteworthy that phenylbutyrate reduced the intracellular accumulation of human preproparathyroid hormone exhibiting the C18R substitution in the H-region of the signal sequence and protected cells against ER stress linked apoptosis (45). Pharmacological treatment of AI in the deciduous dentition would require delivering a drug to the developing foetus *in utero*. Phenylbutyrate is contraindicated during pregnancy but it is licensed for use in neonates and children up to 18 years of age to treat urea cycle disorders where it is given in large doses to act as a nitrogen scavenger to treat hyperammonaemia (by providing an alternative pathway for ammonia secretion) (47). Pharmaceutical intervention based on phenylbutyrate would therefore only be useful to rescue the permanent dentition. In addition, the fact that we observed post eruptive mineralization of affected enamel in *Enam^p.^*^S55I^ heterozygotes suggests that enamel in some forms of AI could benefit from strategies designed to increase/accelerate post-eruptive maturation in humans. The ability to use mouse models to understand AI mechanism in this way coupled with future genetic screening will provide clinicians with information upon which clinical management of their patients’ condition can be predicated.

## Materials and Methods

### Animals

The mutant mouse line M100395 (*Enam^p.^*^S55I^) was obtained from RIKENGSC (http://www.gsc.riken.jp/Mouse/) and maintained on a C57Bl/6J genetic background. All procedures were performed in accordance with the UK Animals (Scientific Procedures) Act, 1986.

### Human tissue samples

An exfoliated human primary incisor affected by AI linked to an *ENAM^p.L31R^* mutation was collected in the UK with the approval of the relevant Research Ethics Committee (reference 13/YH/0028). Unaffected control primary incisors were obtained from the Skeletal Tissues Research Bank (School of Dentistry, University of Leeds) and used with ethical approval and patient consent.

### Histological and immunofluorescence analysis of mouse enamel organ and developing enamel

Mandibles from 2- to 3-month-old heterozygous and homozygous *Enam^p.^*^S55I^ mutant and wild-type mice (*n* = 4 in each category) were dissected following cervical dislocation and fixed in 4% paraformaldehyde in PBS, pH 7.4, at room temperature for 48 h. Following fixation, the mandibles were decalcified in 0.5 M EDTA, dehydrated through a graded ethanol series, cleared in chloroform, embedded as hemi-mandibles in paraffin wax, sectioned and stained with haematoxylin and eosin. Sections were examined using a DMRB microscope (Leica) with SpotTM digital camera and associated software (RTKE/SE Diagnostic Instruments Inc.). Immunofluorescence analysis was performed on mandibles prepared as above using antibodies raised against activated caspase 3 (Abcam, Cambridge, UK), amelogenin FL191, (Santa Cruz Biotechnology, Santa Cruz, CA), ameloblastin (C17, Santa Cruz Biotechnology, Santa Cruz, CA), enamelin, phosphohistone H3 (Abcam, Cambridge, UK), keratin 14 (Abcam, Cambridge, UK) and GRP-78 (Abcam, Cambridge, UK).

Primary antibodies were detected using biotinylated secondary antibodies (Vector laboratories) followed by Cy-3-conjugated streptavidin (Sigma, Poole, UK) or an AlexaFluor488-conjugated secondary antibody (Abcam, Cambridge, UK) and the sections were mounted in fluorescence mountant containing DAPI (Vector laboratories).

### Quantitative reverse transcriptase-PCR of mouse enamel organ mRNA

For quantitative RT-PCR analysis total RNA was extracted using the RNeasy kit (Qiagen, Crawley, UK) from micro-dissected secretory-stage enamel organs (4 wild-type male, 4 *Enam^p.^*^S55I^ heterozygous and 4 *Enam^p.^*^S55I^ homozygous mice) and quantified using a NanoDrop 2000 spectrophotometer (ThermoShandon, Runcorn, UK), then reverse transcribed to complementary DNA. Quantitative RT–PCR was performed according to the manufacturer's instructions on a StepOne Plus machine using SYBR Green master mix (Life Technologies, Paisley, UK) and analysed using the ΔΔ-Ct method, normalized to β-actin (levels of which were consistent in all samples). Results were analysed using a one-tailed Mann-Whitney U test. The following primer pairs were used for the analysis: *Hspa5* 5’-ATCTTTGG TTG CTTG T CG CT-3’, 5’- ATGAAGGAGACTGCTGAGGC-3’; *Ddit3* 5’- GA CCA GGT TCTGCTTTCAGG-3’, 5’- CAGCGACA GAGCCA GA AT A A-3’; *Actb* 5’-CTA AGG CCAACCGTGAAAAGAT-3’, 5’- GCCTG GA T GGC TAC GTA C  ATG-3’.

### Identification of ENAM mutation in human AI

Genomic DNA was obtained from saliva collected using Oragene® DNA Sample Collection kits (DNA Genotek, Ottawa, ON, Canada) as detailed in the manufacturer’s instructions.

Three micrograms of genomic DNA was prepared for whole-exome sequencing using the SureSelect All Exon v5 XT reagent (Agilent Technologies, Santa Clara, CA, USA). Sequencing was performed on an Illumina Hi-Seq 2500 sequencing platform (Illumina, San Diego, CA, USA), using a 100 bp paired-end protocol. Fastq files were aligned to the human reference genome (GRCh37) using Novoalign software (Novocraft Technologies, Selangor, Malaysia). The resulting alignment was processed in the SAM/BAM format using the SAMtools, Picard (http://picard.sourceforge.net) and GATK programs to correct alignments around indel sites and mark potential PCR duplicates (48,49).

Indel and single-nucleotide variants were called in the VCF format using the Unified Genotyper function of the GATK program. Using the dbSNP database at NCBI, any variants present in dbSNP142 with a minor allele frequency (MAF) ≥1% were then excluded and the remaining variants were annotated using in-house software freely available at http://sourceforge.net/projects/vcfhacks/. Variants in genes known to cause AI were prioritized for investigation.

### PCR and sanger sequencing

The variant was confirmed and segregation was tested in all available family members. Primer sequences can be found in Supplementary Material, Table S2. PCR mastermix HotShot Diamond (Clent Life Science, Stourbridge, UK) was used to amplify sequences. Sanger sequencing was performed using the BigDye Terminator v3.1 kit (Life Technologies, Carlsbad, CA, USA) according to manufacturer’s instructions and resolved on an ABI3130xl sequencer (Life Technologies). Results were analysed using SeqScape v2.5 (Life Technologies).

The *ENAM* variant identified in this study has been submitted to the Leiden Open Variant Database at http://dna2.leeds.ac.uk/LOVD/ variant ID: ENAM000018 and ClinVar, accession number SCV000328938.

### Transmission electron microscopy

Two-month-old mouse mandibles (3 wild-type male, 3 *Enam^p.^*^S55I^ heterozygous and 3 *Enam^p.^*^S55I I^ homozygous mice) were dissected following cervical dislocation and fixed in 2% paraformaldehyde/2% glutaraldehyde prepared in 0.1 M cacodylate buffer containing 0.15 M sucrose and 2 mM calcium chloride (pH 7.3) at 4 °C overnight. Hemi-mandibles were demineralized as above and dissected axially into posterior, medial and anterior portions. Following dissection, the samples were washed with cacodylate buffer, post-fixed in 1% osmium tetroxide, dehydrated through a graded ethanol series, cleared in propylene oxide and embedded in Epoxy resin (100 resin, Agar Scientific Ltd, Stanstead, UK)). Ultrathin sections were contrasted with uranyl acetate and lead citrate, and examined on a Philips model 400 transmission electron microscope.

### Scanning electron microscopy

Three hemi-mandibles from 2- to 3-month-old heterozygous and homozygous *Enam^p.^*^S55I^ mutant and wild-type mice were dissected following cervical dislocation and fixed in 4% paraformaldehyde in PBS, pH 7.4, at room temperature for 48 h. Erupted incisors or mandibles were cut transversely with a scalpel and the cut surfaces polished using1000 grit wet and dry carborundum paper followed by final polishing with 12 000 grit nail file to obtain transverse sections through either the erupted or unerupted incisor enamel. Smear layers were removed by etching the ground surface in 30% phosphoric acid for 20 s followed by thorough rinsing in excess de-ionized water. Teeth were dried overnight under vacuum and sputter coated with gold. Specimens were observed using a Hitachi S-3400N scanning electron microscope (in secondary electron imaging mode) operated at an accelerating voltage of 20 kV and an emission current of 80 µA.

An exfoliated human primary incisor affected by AI linked to an *ENAM^p.L31R^* mutation was fractured along the buccal-lingual mid line. The fractured surface was polished, etched, sputter coated and examined by SEM as described above.

### Micro X-ray computed tomography

Three hemi-mandibles from 2- to 3-month-old heterozygous and homozygous *Enam^p.^*^S55I^ mutant and wild-type mice were dissected and fixed as described for scanning electron microscopy followed by mounting in sealed polypropylene micro tubes (to prevent drying out) together with hydroxyapatite standards. Micro X-ray computed tomography was carried out using a Skyscan 1172 CT scanner (Bruker, Kontich, Belgium). Scans were obtained using a tube voltage of 75 kV at a constant power of 10 W. Image pixel resolution was 8–10 µm and a 0.5 mm aluminium filter was used to reduce beam hardening. The scanner was used in oversize scan mode which allowed hydroxyapatite standards to be imaged along with each individual sample for mineral density calibration purposes. The standards used had relative densities of 0.25, 0.75 (Bruker, Kontich, Belgium) and 2.9 (Himed, Old Bethpage, USA). Projection images were reconstructed using Recon software (Bruker, Kontich, Belgium). Quantitative mineral density maps were generated using ImageJ software (http://imagej.nih.gov/ij/) and 3D rendered videos produced using CTVox software (Bruker, Kontich, Belgium).

The exfoliated human AI tooth and a corresponding healthy control tooth along were scanned as described above except the tube voltage was increased to 100kV and the aluminium and copper filter were engaged to reduce beam hardening.

## Supplementary Material

Supplementary Material is available at *HMG* online.

## Supplementary Material

Supplementary Data
