# Supplementary material for: Amelogenesis imperfecta caused by N-terminal enamelin point mutations in mice and men is driven by endoplasmic reticulum stress
Source: Hum Mol Genet. 2017 Mar 11;26(10):1863–76. doi: 10.1093/hmg/ddx090 (PMC5411757; doi:10.1093/hmg/ddx090)
Supplement: Supplementary Data [file ddx090_Supp.zip › Notes for Supplemental Video.pdf]

Supplemental video file: Illustrative video of rendered µCT images comparing the Enam^{S55I} heterozygous phenotype (left) to wild type (right). Using the molar teeth as reference points the delay in the start of secondary mineralisation in the affected incisor is clear.
