# Supplementary material for: Amelogenesis imperfecta caused by N-terminal enamelin point mutations in mice and men is driven by endoplasmic reticulum stress
Source: Hum Mol Genet. 2017 Mar 11;26(10):1863–76. doi: 10.1093/hmg/ddx090 (PMC5411757; doi:10.1093/hmg/ddx090)
Supplement: Supplementary Data [file ddx090_Supp.zip › Supplemental Figure 1.pdf]

# Figure S1

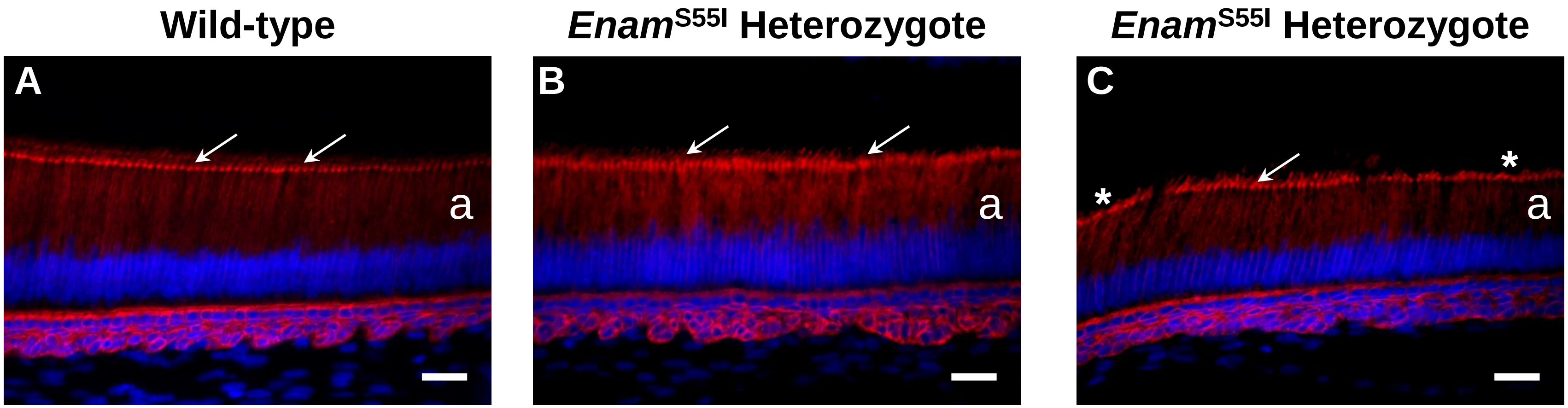


**Figure S1**. Tomes' process disorganisation in *Enam*$^{S55I}$ heterozygous mice as shown by keratin-14 immunohistochemistry. **(A)** In wild-type mice, intense, regularly repeating keratin-14 immunoreactivity, corresponding to the terminal web, can be seen at the apices of the secretory ameloblasts (a) and the less intensely immunolabelled Tomes' processes can be seen to project from the ameloblasts at an angle of approximately 45° (arrows). **(B)** During the early secretory stages of amelogenesis in *Enam*$^{S55I}$ heterozygous mice the ameloblast (a) terminal web region is more diffuse and there are fewer and less well organised Tomes' processes when compared to wild-type mice (arrows). **(C)** At a later stage of enamel matrix secretion in *Enam*$^{S55I}$ mice the Tomes' processes continue to be disorganised and sparse (arrows) and, as they retract, ruptures between the ameloblasts and the enamel matrix appear (asterisks). Scale bars: 25 µm.
