# Supplementary material for: Amelogenesis imperfecta caused by N-terminal enamelin point mutations in mice and men is driven by endoplasmic reticulum stress
Source: Hum Mol Genet. 2017 Mar 11;26(10):1863–76. doi: 10.1093/hmg/ddx090 (PMC5411757; doi:10.1093/hmg/ddx090)
Supplement: Supplementary Data [file ddx090_Supp.zip › Supplemental Figure 2.pdf]

Figure S2

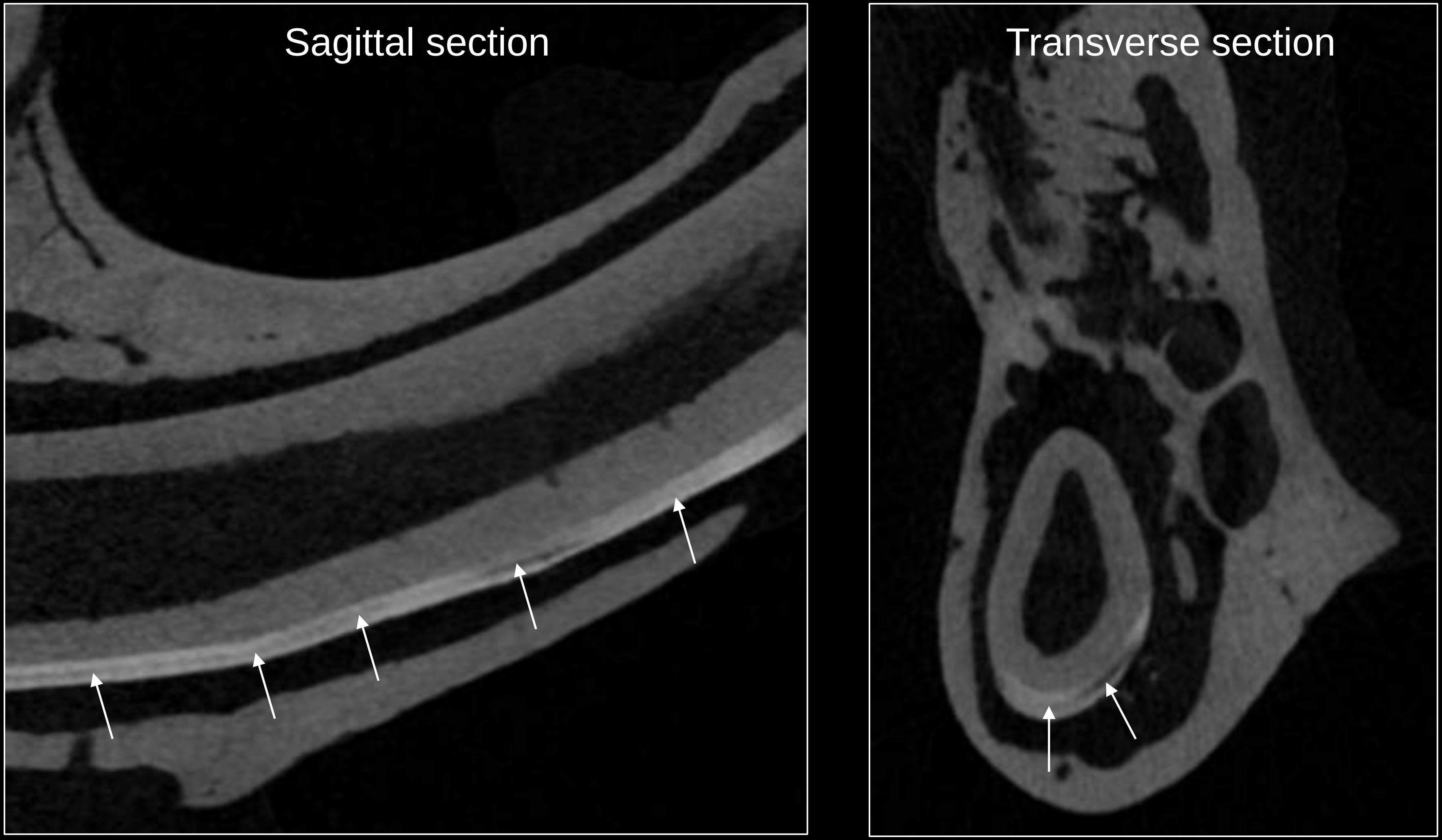

μCT images showing the hypomineralized boundary between the structurally normal inner enamel and the abnormal outer enamel. This discontinuity may represent a fracture plane that is responsible for the rapid loss of the outer enamel layer following eruption of the incisor into the mouth.
