# Supplementary material for: Amelogenesis imperfecta caused by N-terminal enamelin point mutations in mice and men is driven by endoplasmic reticulum stress
Source: Hum Mol Genet. 2017 Mar 11;26(10):1863–76. doi: 10.1093/hmg/ddx090 (PMC5411757; doi:10.1093/hmg/ddx090)

Figure S3

Pedigrees and clinical phenotype of families with heterozygous ENAM c.92C>T, p.(L31R) variant.

Individuals from whom DNA was available for segregation analysis are labelled. The arrow denotes the individual in each family whose DNA was subjected to whole exome sequencing. The clinical images illustrate hypoplastic AI in primary and permanent teeth.

(A) Upper occlusal view of mixed primary and permanent dentition with poor oral hygiene (plaque deposits). Labial view of the upper right permanent central incisor [11] is inset in the top right hand corner. The left sectional panoramic radiograph confirms that very little enamel is present.

(B) Anterior and lower occlusal views of a mixed dentition that includes the permanent incisor and first molar teeth. Note: post eruptive enamel loss on the upper left permanent central incisor [11] and infraoccluded lower left second primary molar [55].

(C) Upper occlusal and lower occlusal views of the permanent dentition. Note: the labial surfaces of the upper and lower anterior teeth have been restored with tooth coloured restorative material and the first permanent molars restored with gold onlays.

(D) Anterior view of the primary dentition and an occlusal view of the upper right teeth: primary canine [53]; primary first and second molars [54, 55] and permanent first molar [16].

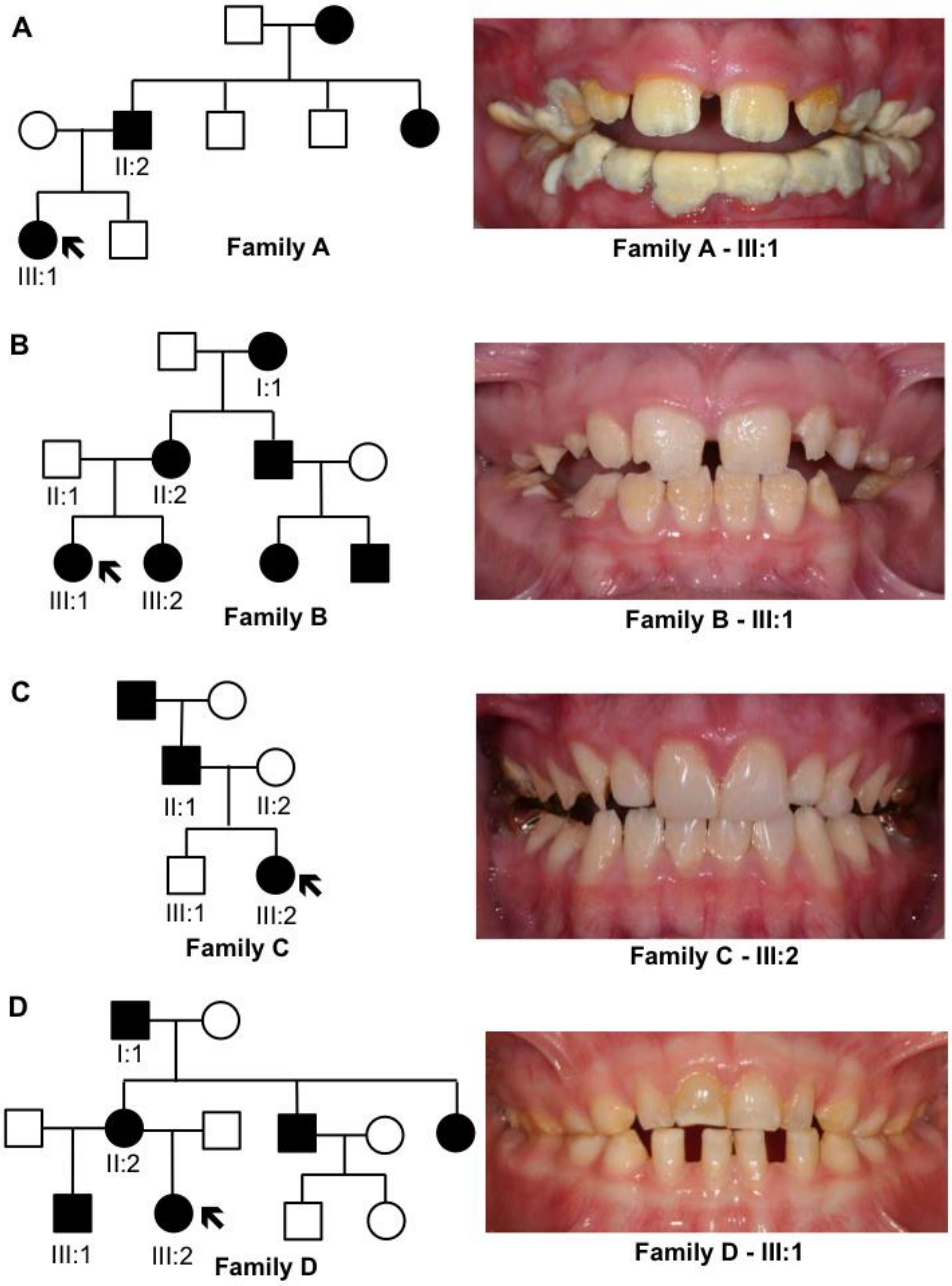

Supplement: Supplementary Data [file ddx090_Supp.zip › Supplemental Figure 3.pdf]
