# Supplementary material for: Amelogenesis imperfecta caused by N-terminal enamelin point mutations in mice and men is driven by endoplasmic reticulum stress
Source: Hum Mol Genet. 2017 Mar 11;26(10):1863–76. doi: 10.1093/hmg/ddx090 (PMC5411757; doi:10.1093/hmg/ddx090)
Supplement: Supplementary Data [file ddx090_Supp.zip › Supplementary tables 1 and 2.pdf]

Supplementary Table 1

Genomic variant (GRCh37)	Transcript variant	Predicted amino acid variant	SIFT[a]	Polyphen-2[b]	Mutation Taster[c]	CADD v1.3[d]	Grantham score[e]
**4:71497424T>G**	**c.92T>G**	**p.L31R**	**Damaging (0)**	**Probably damaging (0.999)**	**Disease causing (0.984)**	**32**	**102**

Table 1 Pathogenicity scores for c.92T>G variant identified in *ENAM.*
Summary of bioinformatics analyses undertaken to predict the pathogenic nature of the variant identified in *ENAM* in Family 1 with AI. For each variant, its predicted pathogenic effect and conservation are calculated using a variety of pathogenicity prediction and conservation score software. SIFT and Mutation Taster annotations were based on the relevant Ensembl transcript, Polyphen-2 annotations were based on the relevant RefSeq protein.
ENAM: Ensembl transcript: ENST00000396073 or RefSeq protein NP_114095.2.
[c] SIFT, http://sift.jcvi.org/ [Ng, P.C. et al. (2003). Nucleic Acids Res. 31, 3812-4];
[d] PolyPhen2, http://genetics.bwh.harvard.edu/pph2/ [Adzhubei, I.A. et al. (2010). Nat. Methods 7, 248-9];
[e] Mutationtaster, http://www.mutationtaster.org/ [Schwarz, J.M. et al. (2010). Nat. Methods 7, 575-6];
[f] Combined Annotation Dependent Depletion (CADD) v1.3, http://cadd.gs.washington.edu/info [Kircher, M. et al. (2014) Nat. Genet. 46, 310-5];
[g] Grantham Score [Grantham, R. (1974) Science 185, 862-4]

Supplementary Table 2

Gene	Exon	Forward primer (5′-3′)	Reverse primer (5′-3′)	Size (bp)
ENAM	3-4	GCTAGTACTTAGATAAGTGCAGAGTGC	GACAATTTTCCAATATTCTCCTTTT	388

Table 2 Primer sequences for Sanger sequencing of the *ENAM* variant identified by WES**.**
